# Breast Pseudoaneurysm Following a Motor Vehicle Collision

**DOI:** 10.7759/cureus.64906

**Published:** 2024-07-19

**Authors:** Alan Y Xu, Mariam Hanna

**Affiliations:** 1 Department of Radiology, University of Florida College of Medicine, Gainesville, USA

**Keywords:** traumatic breast injury, ultrasound, pseudoaneurysm, breast, hematoma

## Abstract

A breast pseudoaneurysm (PSA) is a rare complication, with most cases reported following breast procedures. There are few reported cases of breast PSAs following blunt trauma. We report a rare case of right breast PSA in a 67-year-old female following a motor vehicle collision. The PSA was managed with a simple external pressure dressing over the breast and was found to have spontaneously thrombosed on a follow-up visit. Breast PSAs may be more common than expected due to their ability to spontaneously resolve and should be considered in patients presenting with a pulsatile breast mass or injury to the breast, especially when accompanied by a history of recent breast trauma or procedure.

## Introduction

A pseudoaneurysm (PSA) is a hematoma that communicates with the arterial lumen and contains flowing blood but lacks the three layers of the arterial wall, which distinguishes it from a true aneurysm [1]. Breast PSAs are a rare complication commonly associated with interventional breast procedures such as core-needle biopsies. Spontaneous breast PSA formation is rare and associated with underlying atherosclerotic disease and anticoagulation [2]. They can also occur after blunt trauma, and to the best of our knowledge, there are three cases of breast PSAs due to blunt trauma reported in the English literature [3-5]. We report a rare case of a right breast PSA in a woman following blunt trauma from a motor vehicle collision.

## Case presentation

**Figure 1:**
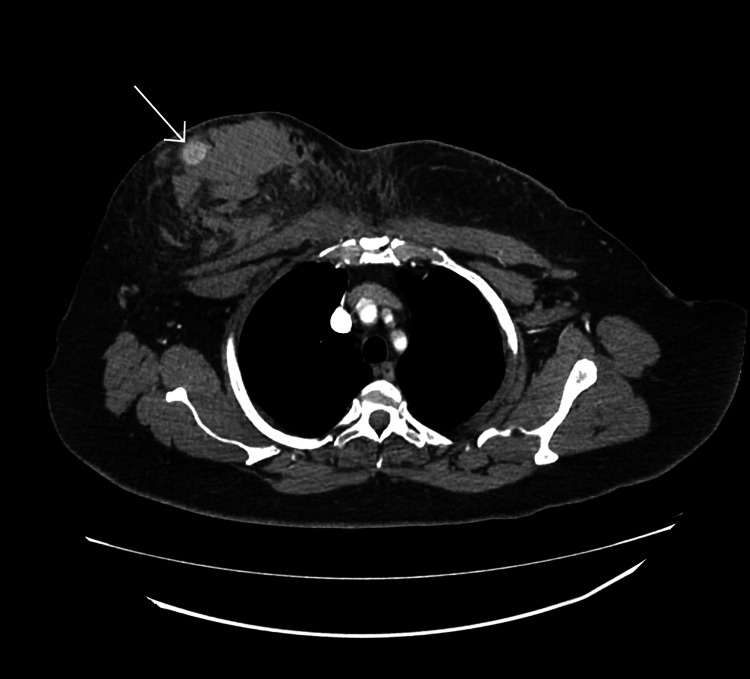
Axial CT image (late arterial phase) showing a well-defined, hyper-dense, right breast lesion with surrounding non-enhancing soft tissue lobulated density and soft tissue edema CT: computed tomography

**Figure 2:**
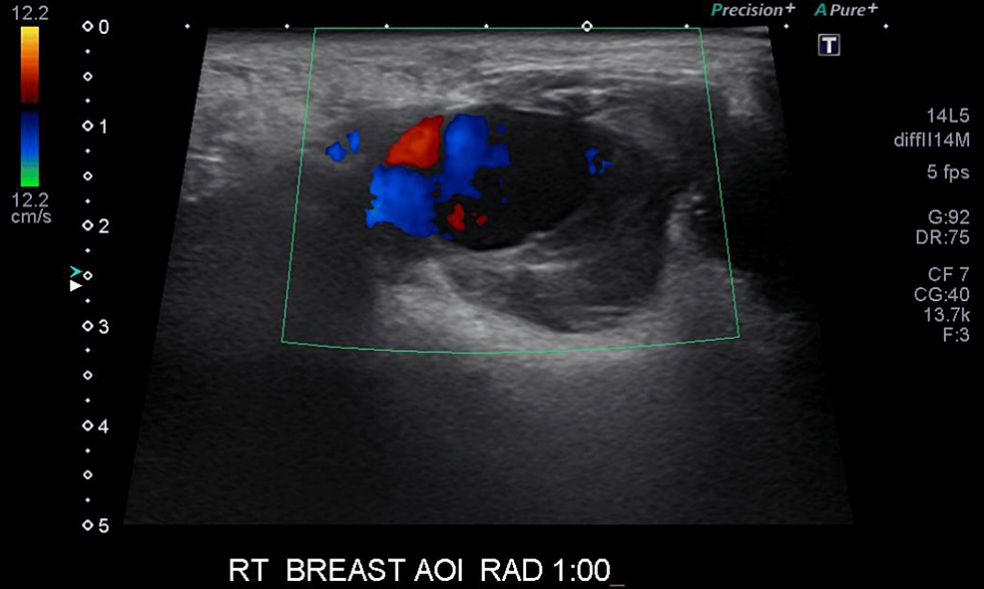
A single-spot image of gray-scale ultrasound with color Doppler analysis demonstrates bidirectional blood flow, or the Yin-Yang sign, consistent with a pseudoaneurysm

A 67-year-old female presented to the emergency department with a four-day history of worsening chest pain, bruising, and swelling following a motor vehicle collision in which she was a restrained driver and accelerated into another vehicle from the stopped position. She had a past medical history of high cholesterol, hypertension, hypothyroidism, and chronic kidney disease and was not on blood anticoagulants. A physical exam revealed bilateral bruising of the breast and tense hematoma of the right breast with overlying abrasion. There was no active bleeding or expanding hematoma. The patient was advised to apply constant pressure dressing to her right breast. A breast ultrasound was ordered after a 1.5 cm hyperdense, well-circumscribed ovoid lesion was noted superficially within the hematoma on computed tomography (CT) cross-sectional imaging (Figure 1). Breast ultrasound revealed a superficial PSA of the right breast measuring 16.7 mm at the outer 9:00 position with a perforator vessel from the intercostal artery tracking superomedially and surrounding hematoma (Figure 2).

**Figure 3:**
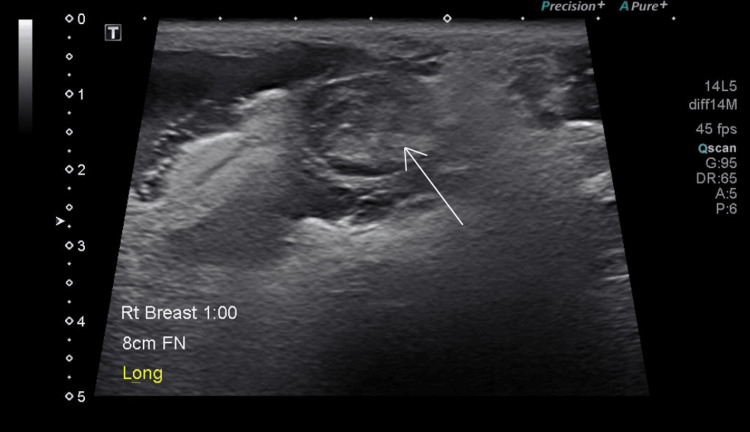
Single gray-scale ultrasound demonstrating thrombosed pseudoaneurysm There is surrounding hematoma and blood products.

The patient was evaluated by a surgical team a week later, and a repeat ultrasound revealed thrombosis of the right breast PSA with a persistent large, symptomatic hematoma. She underwent a mastotomy and 200 cc of hematoma was evacuated. She tolerated the procedure with no complications. On follow-up a month later, her surgical sites were clean, dry, and healing well, and the bruising had resolved with a small residual hematoma (Figure 3).

## Discussion

Breast PSAs should be suspected in patients presenting with palpable pulsatile breast masses, hematomas, or extensive bleeding of the breasts, especially following a recent history of trauma or biopsy [2]. Hematomas are the most common post-procedural complications and typically occur immediately or within days of biopsy [6]. They can also form after blunt force trauma to the breast in cases such as motor vehicle accidents and falls [6]. These patients may also present with extensive cutaneous bruising over the affected area [1]. Ultrasound is the preferred imaging modality for evaluating breast PSAs. This diagnosis can be easily performed with color Doppler ultrasound with an accuracy of more than 95% [7]. PSAs demonstrate a characteristic swirling flow pattern, or the “Yin-Yang” sign, on color Doppler ultrasound [1,8].

While the natural history of PSAs is not well understood, many PSAs spontaneously thrombose [8]. The likelihood of spontaneous thrombosis is influenced by PSA size, length of the neck, and anticoagulation status of the patient [8]. In general, larger PSAs, wider necks, and increased coagulation result in decreased likelihood of spontaneous thrombosis [8]. The first-line treatment option for breast PSAs is ultrasound-guided manual compression. Ultrasound imaging can guide manual compression of the neck of the PSA for 30-60 minutes and can be effective for small PSAs such as those <2 cm (such as the one seen in this patient) [1]. This technique has a lower risk for occlusion of the native vessel and avoids the systemic effects of alternative treatments like thrombin injection [8]. Immediate color Doppler ultrasound imaging should be performed later to confirm the resolution of flow. Follow-up ultrasound imaging two to seven days later should also be performed to ensure continued thrombosis [1]. Based on only a few case reports, manual compression is thought to have a lower success rate when managing PSAs of the breast compared to other locations in the body. Suggested reasons for this include a higher incidence of wide-neck PSAs, owing to the use of large-gauge biopsy devices, and flexibility of breast tissue, which disrupts early thrombus formation and makes it more difficult to apply adequate pressure [2].

In cases where compression fails, direct injection with thrombin, percutaneous injection with alcohol, or embolic agents can be used to treat PSAs [1,8]. In rare circumstances, where other treatment options have been exhausted, surgical intervention may be needed. The patient described in this case had a relatively small and superficial PSA that resolved with only external pressure dressing. Breast PSAs may be more common than reported owing to their tendency to spontaneously resolve, especially when small and treated early [2]. The superficial location of our reported breast PSA likely allowed for better compression of the PSA against the chest wall.

## Conclusions

While PSAs are regarded as iatrogenic complications that physicians are aware of after performing invasive procedures, they should also be considered in the context of traumatic injuries. Trauma-induced breast PSAs can present with symptoms such as chest pain, bruising, swelling, and hematoma. This case report highlights a rare, non-iatrogenic incidence of breast PSA that resolved on its own when managed early with manual compression. While breast PSAs may spontaneously resolve, it is important to emphasize early detection and intervention of PSAs, as this will ultimately reduce stress and discomfort in patients and produce better outcomes.
